# Genetic signatures of parental contribution in black and white populations in Brazil

**DOI:** 10.1590/S1415-47572009005000001

**Published:** 2009-01-10

**Authors:** Vanderlei Guerreiro-Junior, Rafael Bisso-Machado, Andrea Marrero, Tábita Hünemeier, Francisco M. Salzano, Maria Cátira Bortolini

**Affiliations:** Departamento de Genética, Instituto de Biociências, Universidade Federal do Rio Grande do Sul, Porto Alegre, RSBrazil

**Keywords:** admixture dynamics, mtDNA, Y-chromosome markers

## Abstract

Two hundred and three individuals classified as white were tested for 11 single nucleotide polymorphisms plus two insertion/deletions in their Y-chromosomes. A subset of these individuals (n = 172) was also screened for sequences in the first hypervariable segment of their mitochondrial DNA (mtDNA). In addition, complementary studies were done for 11 of the 13 markers indicated above in 54 of 107 black subjects previously investigated in this southern Brazilian population. The prevalence of Y-chromosome haplogroups among whites was similar to that found in the Azores (Portugal) or Spain, but not to that of other European countries. About half of the European or African mtDNA haplogroups of these individuals were related to their places of origin, but not their Amerindian counterparts. Persons classified in these two categories of skin color and related morphological traits showed distinct genomic ancestries through the country. These findings emphasize the need to consider in Brazil, despite some general trends, a notable heterogeneity in the pattern of admixture dynamics within and between populations/groups.

## Introduction

The great maritime expeditions and colonial expansion by Europeans during the 15^th^ and 16^th^ centuries considerably changed their history and those of the colonized peoples. The cultural impact of colonization has been known for a long time, but only more recently have its genetic aspects have been investigated. Genetic studies of Brazilian populations have shown, for example, that the extent of admixture among Europeans, Africans and Native Americans is higher than perceived by phenotypic characteristics, and that marriages during the Colonial Era were mainly asymmetric, with most of the Y-chromosomes present in contemporary white Brazilian populations being of European origin, but a significant portion of the mtDNA lineages being of Amerindian or African origin (Alves-Silva *et al.*, 2000; Carvalho-Silva *et al.*, 2001). However, marked regional differences have been reported for white Brazilian populations, indicating that there can be considerable variation in the general trends (Callegari-Jacques *et al.*, 2003; Marrero *et al.*, 2005, 2007a; Zembrzuski *et al.*, 2006). DNA studies with urban black populations have been less common but have also identified important regional differences (Bortolini *et al.*, 1999; Hünemeier *et al.*, 2007; Gonçalves *et al.*, 2008).

In this work, we investigated the genetic background of people classified as white or black living in the same city (Porto Alegre, the capital of the southernmost state of Brazil). Our results reveal details about the history of Porto Alegres human population and indicate the extent of genetic admixture, as well as the nature of this process within and between these two major color groups in Brazils four distinct geographical regions. These findings also show how it is possible to discover details of the ancestral history of a contemporary population based on its current genetic characteristics.

## Subjects and Methods 

### Population samples and DNA extraction

The history of Porto Alegre (30°5' S, 5°10' W), the capital of the Brazilian state of Rio Grande do Sul, dates from 1730 when Jerônimo de Ornellas, an immigrant from Madeira Island, received a large plot of land (*sesmaria*) in this area from the Portuguese crown. The little village that was subsequently formed showed significant population growth only after the arrival of ~500 couples from the Azores archipelago (another Portuguese colony), from 1752 to 1754 (Flores, 1996). The importance of the Azorian migration to Rio Grande do Sul, especially Porto Alegre, is illustrated by Laytano (1974), who stated that in 1780 about 55% of the 18,000 inhabitants of the town were Azorian or of Azorian-descendent. The pre-Columbian inhabitants, the Guarani Indians, were displaced, although in other regions of the state they, as well as other natives, were still present (Noelli *et al.*, 1997). Subsequent waves of European migration, especially from Portugal, Spain, Italy and Germany, contributed to the growth of Porto Alegre and nearby cities. The presence of African slaves is also mentioned in historical records (Nogueról, 2005), although their proportion relative to the total population is unknown. Currently, the urban complex formed by the capital and neighboring cities has 3,152,596 inhabitants, 7% and 88% of whom are classified as blacks (*pretos*, in Portuguese) and whites (*brancos*), respectively (Brazilian Institute of Geography and Statistics-IBGE, 2000). In Brazil, skin color rather than ancestry is used to define an equivalent to “race”, and in the present study the word “black” will be used to refer to *pretos* and any person identified and/or self-identified with some other term that suggests African ancestry, such as *mulato* or *pardo.* “White” will be used to define those who, based on their physical traits and information, show no admixture with non-Europeans.

Two hundred and three unrelated individuals living in Porto Alegre and its metropolitan region, phenotypically classified as white by the interviewer of the research group, were studied. DNA was extracted from whole blood according to Lahiri and Nurnberger (1991) or from saliva using QIAamp® kits (QIAGEN), according to the manufacturer's instructions. This investigation was approved by the Brazilian National Ethics Commission (CONEP Resolution no. 1333/2002).

### Y- chromosome markers

Hünemeier *et al.* (2007) recently presented and discussed the African portion of the Y-chromosomes and mtDNA lineages of a sample of 107 blacks from the same city. Here, we investigated additional Y-chromosome polymorphisms (see below) to characterize the non-African portion of the Y-chromosomes of this sample, and also considered the mtDNA lineages classified as Amerindian or European. This procedure standardized the set of uniparental markers investigated in the two samples, thereby allowing comparative analyses.

Two hundred and three white men were genotyped for 13 binary loci (11 single nucleotide polymorphisms or SNPs: 92R7, M3, M242, M9, M17, M170, M173, M213, M269, SRY2627, M2, and two insertion/deletions, Yap *ALU* and 12f2) located in the non-recombinant region of the Y-chromosome (NRY). These markers define well the most common and widespread African, European and Native American Y-chromosomes. Genotyping was done according to the hierarchical order of markers presented by Jobling and Tyler-Smith (2003) using the primers and conditions described in Hammer and Horai (1995), Underhill *et al.* (1996, 2001), Hurles *et al.* (1999), Thomas *et al.* (1999), Rosser *et al.* (2000), Bortolini *et al.* (2003), Flores *et al.* (2003), Montiel *et al.* (2005), and Kayser *et al.* (2005). Haplogroups P*(xQ3,R1), Q3*, K(xP), Q*(xQ3), R1a1*(xDE), I*(xP,K,J), R1*(xR1a1,R1b3), F(xI,J,K,P), R1b3*(xR1b3f), R1b3f, E3a*, DE*(E3a) and J* were established using the markers indicated above in the order given. According to Jobling and Tyler-Smith (2003), designations such as P*(xQ3, R1) indicate partial typing of markers in a haplogroup and, in the present case, describes all chromosomes in clade P* except Q3* and R1*. We also investigated 11 of the biallelic loci indicated above (92R7, M3, M242, M9, M17, M170, M173, M213, M269, SRY2627 and 12f2) to characterize the non-African portion of the Y-chromosomes in the black sample previously studied by Hünemeier *et al.* (2007).

### Mitochondrial DNA

The first hypervariable segment (HVS-I) of the mtDNA control region was sequenced in a subset of 172 white individuals using primers and conditions previously described by Marrero *et al.* (2005). The sequence reactions were run in an automated ABI 310 sequencer. Both DNA strands were sequenced from positions 16050 to 16391, since this is the region for which most of the comparative information is available. Individuals with the “C-stretch” between positions 16183-16193, which is caused by the 16189C substitution, were re-sequenced in each direction so that each base was determined twice.

The sequences were checked manually, and validated by using the CHROMAS LITE 2.0 program. Alignment relative to the revised Cambridge Reference Sequence (Andrews *et al.*, 1999) was done using the BIOEDIT software (Hall, 1999). The filtering procedure described by Bandelt *et al.* (2002) was used to check the quality of the sequences and eliminate artifacts introduced during sequencing and editing. After filtering, the relationships between the lineages were examined with the NETWORK 4.2.0.0. program using the median-joining algorithm (Bandelt *et al.*, 1999). Weight networks showing star tree patterns, together with other criteria such as those suggested by Yao *et al.* (2004), ensured that the data were essentially free of artifacts. The HVS-I sequences were classified into haplogroups according to recommended criteria (Bandelt *et al.*, 2002; Salas *et al.*, 2002, 2004; Kivisild *et al.*, 2002; Torroni *et al.*, 2006).

### Data analysis

Y-SNP and mtDNA haplogroup frequencies were obtained by counting. Estimates of parental continental contributions in the paternal and maternal data sets were obtained directly since the major haplogroups of mtDNA and Y-SNP are geographically specific. Population relationships were estimated through F_ST_ genetic distances using Arlequin version 3.01 (Schneider *et al.*, 2000; Excoffier *et al.*, 2005) and their statistical significance was assessed by permutation using 10,000 runs.

## Results and Discussion

### Y-chromosome biallelic polymorphisms (SNPs)

Based on the Y-chromosome distribution (Table 1), the most frequent haplogroup in Porto Alegre whites was R1b3* (51%), which was also the most common haplogroup found in another Brazilian city (Rio de Janeiro), as well as in the Azores and Portugal. The frequency of this haplogroup was lower (~13%) in Porto Alegre blacks.

To test the hypothesis of random haplogroup distribution among populations we computed F_ST_ values based on the major hierarchical clustering for these analyses. There were no significant differences between Porto Alegre whites and those of the Azores, Portugal or Spain (F_ST_ = -0.00162, p = 0.6126; F_ST_ = 0.00235, p = 0.1622; F_ST_ = 0.00345, p = 0.1261, respectively), although the Porto Alegre white prevalences differ significantly from those of the other European countries considered. The difference between Porto Alegre and Rio de Janeiro was also significant (F_ST_ = 0.0120, p = 0.0090), but when the African (E3a, B*, and DE) and Amerindian (Q* and Q3*) haplogroups are excluded this significance disappeared (F_ST_ = 0.0056, p = 0.1441). Surprisingly, the haplogroups of European origin detected in Porto Alegre blacks were significantly different from those found in whites of the same city (F_ST_ = 0.1562, p = 0.000), and from those observed in Iberian populations. However, since the number of blacks studied was small (30), it is probable that these differences reflect sampling error rather than any phenomenon of the admixture process. The African portion of the Y-chromosomes of this black sample has been described in detail by Hünemeier *et al.* (2007).

### Mitochondrial DNA

Multiple alignment with the reference sequence (Andrews *et al.*, 1999) allowed the identification of 105 mtDNA lineages in white individuals (Table 2). This table also shows the non-African lineages identified in blacks, as well as those shared by the two samples.

For 35 of the 69 European lineages (51%), identical matches were found for data in the literature from countries with an important history of migration to Rio Grande do Sul (Portugal/Azores, Spain, Italy, and Germany; data from Crespillo *et al.*, 2000; Pereira *et al.*, 2000; Mogentale-Profizi *et al.*, 2001; Brehm *et al.*, 2003; Poetsch *et al.*, 2003; Picornell *et al.*, 2005; Pichler *et al.*, 2006). Most of these haplotypes (25; 71%) perfectly matched more than one of the European populations. Four (lineages #12, #13, #38 and #68), three (#36, #62 and #63), two (#2 and #38), and one (#14) of the haplotypes found in Porto Alegre (Table 2) exclusively matched those found in the Azores, Portugal, Spain, and Italy, respectively. Only two lineages of European origin (#49 and #50) present in Porto Alegre blacks were not observed in whites from the same city or in any of the European populations considered here. As with the Y markers, the mtDNA data also revealed that the Azorian presence in Porto Alegre was clearly detectable regardless of the demographic and cultural changes that occurred after the initial foundation/colonization of the city.

The same analysis with the 14 lineages of African origin found in the white sample revealed that 57% of them had perfect matches with those found in the regions of slave importation to Brazil (West, West-Central, and Southeast Africa; Salas *et al.*, 2002; Plaza *et al.*, 2004; Rosa *et al.*, 2004; Beleza *et al.*, 2005; Coia *et al.*, 2005; Jackson *et al.*, 2005). Three lineages (# 103, #104, and #116) exclusively matched those found in Angola, Cabinda, and Mozambique, countries inhabited by peoples who speak Bantu languages (Salas *et al.*, 2002; Plaza *et al.*, 2004; Beleza *et al.*, 2005). Another lineage (#110) was also probably of Bantu origin since it occurs in Mozambique and in the Bassa ethnic group of Cameroon. Lineage #113 matched one found only in Guinea Bissau, whereas # 106 matched another found in Sierra Leone, both of which are West African countries (Rosa *et al.*, 2004; Jackson *et al.*, 2005). Two lineages (#108 and #109) had a geographical origin that was difficult to define since they occur in all sub-Saharan Africa. Only four African lineages were shared between the black and white Porto Alegre groups, with all four occurring in Africa and/or other Brazilian populations (Salas *et al.*, 2002; Plaza *et al.*, 2004; Rosa *et al.*, 2004; Beleza *et al.*, 2005; Coia *et al.*, 2005; Jackson *et al.*, 2005; Gonçalves *et al.*, 2008).

The four major Amerindian haplogroups were detected among Porto Alegre whites (A = 28%; B = 22%, C = 47%, D = 3%), whereas haplogroup A was absent among blacks (B = 19%, C = 69% and D = 12%). Lineage #87 was the most common in both samples. Since this lineage contained the mutations that defined the C nodal branch, it matched several sequences found in admixed populations from southern Brazil, and in individuals from the Tupian and Jêan tribes; these different matches precluded identification of the precise origin of this lineage (Alves-Silva *et al.*, 2000; Marrero *et al.*, 2005, 2007a,b). On the other hand, the Guarani contribution was clearly detected through the presence of lineages such as #75 (Marrero *et al.*, 2007b). However, most of the Amerindian lineages found in Porto Alegre did not match and/or cluster with Guarani mtDNA sequences. These results support the idea that the present Guarani mitochondrial genome may be a poor representative of that found at colonial times (Marrero *et al.*, 2007a). This finding also suggests that other tribes may have made a more significant contribution, through their women, to the formation of the contemporary admixed Porto Alegre population.

### Ancestral contributions

Table 3 summarizes the continental origins of the two Porto Alegre samples based on the mtDNA and Y-chromosome haplogroups. Although there was a significant introduction of non-European mtDNA sequences (Amerindian = 21%; African = 10%) among whites, Europe was still the major contributor in both genetic systems (mtDNA = 69%; Y-chromosome = 99%). This finding supported the general correspondence between physical appearance and maternal or paternal ancestry at the population level. In contrast, a completely different picture emerged when the black group was considered. Most of the mtDNA sequences (79%) had an African origin, but 56% of the Y-chromosomes were of European origin, while the Amerindian contribution involved both paternal and maternal inheritance.

These results for the black group can be partly explained by the fact that *mulatos* and *pardos*, who showed visible signs of admixture, were included in this sample. However, history and the dynamics of admixture could also have played an important role. In the early centuries of colonization, almost only European men migrated to Brazil, and for different reasons, African males were brought preferentially to Brazil during the slave trade (Mattoso, 1982). This initial demographic asymmetry, and compulsory restrictions to the African male slaves reproduction, determined that the first Brazilians were born mostly from the union between European males and Amerindian or African females. Later, social practices determined that a child with more pronounced physical African features would be considered black, while those with more pronounced European features would be considered white. This situation created ample opportunity for the introduction of African mtDNA lineages and European Y-chromosomes into the white and black segments, respectively (Parra *et al.*, 2003; Gonçalves *et al.*, 2007). A second major European migratory movement during the 19^th^ century that was particularly important for southern Brazil involved couples and families, not just males. This migration resulted in many white persons/populations with complete European genomes, whereas others are phenotypically white but show non-European admixture signs at the genome level (Marrero *et al.*, 2005; Zembrzuski *et al.*, 2006).

To investigate whether these differences between the two skin color groups were peculiar to Porto Alegre or whether they represented a more general tendency, we compiled the literature data for all of the estimates of African, European and Amerindian contributions in black and white Brazilian populations from different geographical regions. Table 4 shows that the pattern described above occurs throughout southern Brazil, but not in other regions of the country. In blacks from the Southeast, for example, the African component predominates in both paternal and maternal data sets, whereas among whites in this region the European contribution is particularly frequent when only Y chromosome markers are considered. Table 4 also show that the whites from the North and South present large differences, basically related to the Amerindian and European women contribution; the extensive admixture between Indians and non-Indians ended in southern Brazil at least 170 years ago, whereas in northern Brazil, especially in the Amazon basin, Amerindian genes are still being introduced into non-native urban and rural populations. The results of these discontinuous *vs.* continuous patterns of gene flow can be seen by comparing the mtDNA results mentioned above with the biparental loci admixture values (21% *vs.* 54% and 13% *vs.* 44% for the Amerindian component in white samples from the South and North, respectively). In contrast, groups or populations identified as white in the Northeast and Southeast had very similar proportions of Amerindian, European and African ancestries in all of the genetic systems investigated. Since European colonization started almost simultaneously in the Northeast and Southeast, this similarity suggests that the dynamics of admixture were similar in both regions.

These striking differences between and within the skin color groups in Brazilian regions can be due to several factors, and some can be the same as those presented for the Porto Alegre case. The type of classification employed to classify the samples in the different studies is also important. Both self and interviewer classifications are full of subjectivities (Salzano and Bortolini, 2002). Distinct admixture dynamics due to historical events and social practices should also be considered. Overall, our results corroborate the idea that, despite some general trends, there is a notable heterogeneity in the pattern of admixture within and among populations/groups in Brazil (Bortolini *et al.*, 1999; Alves-Silva *et al.*, 2000; Marrero *et al.*, 2005). Our findings also highlight the intricacies of past and present patterns of mating in a complex society with a relatively recent multiethnic origin, and the relative instability of phenotypic classifications within this society.

## Figures and Tables

**Table 1:** Y-SNP haplogroup frequencies in Porto Alegre residents classified as white or black compared with individuals from another Brazilian city (Rio de Janeiro) and European populations.

Geographic origin	N	B	DE	E3A	F*	I	J	K*	P*	Q*	Q3	R1*	R1a1*	R1b3*	R1b3f
Brazil															
Porto Alegre whites^a^	203	0.000	0.064	0.005	0.104	0.078	0.108	0.015	0.019	0.000	0.000	0.009	0.054	0.515	0.029
Porto Alegre blacks^a^	54	0.037	0.093	0.259	0.185	0.037	0.037	0.037	0.074	0.037	0.018	0.055	0.000	0.131	0.000
Rio de Janeiro unclassified^b^	127	0.000	0.119	0.079	0.023	0.102	0.071	0.055	ND	ND	0.015	0.000	0.024	0.512	ND
Azores^c^	185	0.000	0.090	0.008	0.083	0.066	0.133	0.017	ND	ND	0.000	0.017	ND	0.57	0.017
Azores^d^	121	0.000	0.130	ND	0.059	0.086	0.086	0.038	0.021	ND	ND	0.560	ND	ND	0.022
Portugal^e^	657	0.000	0.123	0.002	0.057	0.076	0.104	0.019	ND	ND	ND	0.020	ND	0.577	0.022
Spain^f^	380	0.000	0.102	0.005	0.034	0.113	0.110	0.045	0.000	ND	0.000	0.541	ND	ND	0.050
Italy^g^	1060	ND	0.156	0.000	0.116	0.093	0.228	0.035	0.003	ND	ND	0.344	0.025	ND	ND
Germany^h^	1215	ND	0.064	ND	0.043	0.236	0.040	0.032	0.013	ND	ND	0.393	0.179	ND	ND
Poland^h^	913	ND	0.050	ND	0.020	0.173	0.025	0.042	0.003	ND	ND	0.116	0.571	ND	ND

^a^Present study; ^b^Silva *et al.* (2006); ^c^Gonçalves *et al.* (2005); ^d^Montiel *et al.* (2005); ^e^Beleza *et al.* (2006); ^f^Flores *et al.* (2003); ^g^Capelli *et al.* (2006, 2007); ^h^ Kayser *et al.* (2005).

**Table 2:** mtDNA haplotypes and haplogroups in Porto Alegre residents classified as black or white.

Lineage	Number of sequences		HVS-I mutations^a^	Haplogroup^b^	Origin
#	White	Black^c^			
1	22	2	rCRS	H	European
2	4		126	H	European
3	3		153	H	European
4	2		261	H	European
5	2		354	H	European
6	2		311	H	European
7	1		83	H	European
8	1		93	H	European
9	1		114	H	European
10	1		168	H	European
11	1		189	H	European
12	1		240	H	European
13	1		124 354	H	European
14	1		93 304	H	European
15	1		209	H	European
16	1		233	H	European
17	1		189 300 325	H	European
18	1		361	H	European
19	2		162	H1a	European
20	1		304	H5	European
21	1		129 223	I	European
22	2		69 126 192	J	European
23	1		69 126 193 300 309	J	European
24	1		69 126 366	J	European
25	1		69 126 222	J	European
26	2		69 126 145 231 261	J2a	European
27	1		69 93 126 261 274 355	J2a	European
28	1		69 126 261	J2a	European
29	1		69 126 145 172 222 261	J1b1	European
30	1		69 126 145 172 222 261 305T	J1b1	European
31	3		69 126 193 278	J1c	European
32	1		69 111 126	J1c	European
33	1		188 224 311	K	European
34	3	1	224 311	K	European
35	1		83 224 311	K	European
36	1		86 224 311	K	European
37	1	1	93 224 290 311	K	European
38	1		93 224 311	K	European
39	1		129 223 291 298	M or I	European
40	1		129 183C 189 249 311	M1 or U1a	European
41	1		126 145 176G 223 260	N1b	European
42	1		145 176G 223	N1b	European
43	1		126 292 294	T	European
44	1		51 126 294	T	European
45	3		126 163 186 189 284 294	T1	European
46	1		126 254 294 296 304	T2b	European
47	3		126 294 296 304	T2b	European
48	1		126 294 296 304 360	T2b	European
49	1		126 193 294 296 304 357	T2b	European
50		1	126 294 296 311	T2b	European
51		1	126 193 294 296 304	T2b	European
52	2		126 220 292 294	T3	European
53	1		126 163 186 189 294	T3	European
54	1		51 129C 183C 189	U2e	European
55	1		189 319 356	U4	European
56	2		270	U5	European
57	2		192 256 270	U5	European
58	1		192 270	U5	European
59	1		256 270	U5	European
60	4		167 192 256 270 311 318	U5a	European
61	1		189 256 362	U5a	European
62	1		256 270 342	U5a	European
63	2		172 183C 189 219 278	U6a	European
64	1		172 183C 189 278	U6a	European
65	3		153 298	V	European
66	3		298	V	European
67	1		187 298 311	V	European
68	1		291 298	V	European
69	1		189 223 278	X	European
70	3		126 223 278 290 319 362	A	Amerindian
71	1		111 223 290 319 362	A	Amerindian
72	1		111 223 290 319	A	Amerindian
73	1		111 129 223 290 319 362	A	Amerindian
74	1		111 126 223 259 290 319 362	A	Amerindian
75	1		111 223 266 290 319 362	A	Amerindian
76	1		111 223 269 290 319 360 362	A	Amerindian
77	1		92 111 223 290 319 362	A	Amerindian
78	2		178 183C 189 217	B	Amerindian
79	1	1	189 217	B	Amerindian
80	1		178 183C 189 217 311	B	Amerindian
81	1		178 183C 189 217	B	Amerindian
82	1		83 189 217	B	Amerindian
83	1		183C 189 217	B	Amerindian
84	1		189 217 249 312 344	B	Amerindian
85		1	189 217 311 319	B	Amerindian
86		1	189 217 311	B	Amerindian
87	10	5	223 298 325 327	C	Amerindian
88	1		114 123 298 325 327	C	Amerindian
89	1		223 224 298 311 325 327 356	C	Amerindian
90	1		223 270 298 325 327	C	Amerindian
91	1		126 207 223 298 325 327	C	Amerindian
92	1		051 172 223 295 298 325 327 335	C	Amerindian
93	1		185 209 223 327	C	Amerindian
94	1		187 223 290 325	C	Amerindian
95		2	051 223 298 325 327	C	Amerindian
96		1	223 298 325 327 356	C	Amerindian
97		1	051 223 287 298 311 325 327	C	Amerindian
98		1	223 298 325 327 362	C	Amerindian
99		1	223 325 327	C	Amerindian
100	1		223 239 288 325 362	D	Amerindian
101		1	189 223 325 362	D	Amerindian
102		1	223 325 362	D	Amerindian
103	3		148 172 187 188G 189 223 230 311 320	L0a2	African
104	1	3	129 148 168 172 187 188G 189 223 230 278 293 311 320	L0a1	African
105	1		66 129 179 187 189 218 223 230 243 290 311	L0d	African
106	2		111 126 187 189 223 239 270 278 293 311	L1b	African
107	1		83 126 187 189 215 223 264 270 278 293 311	L1b	African
108	1	2	126 187 189 223 264 270 278 293 311	L1b	African
109	1	2	126 187 189 223 264 270 278 311	L1b	African
110	1		129 187 189 223 274 278 293 294 311 360	L1c1	African
111	1		134 187 213 223 265C 274 278 286G 360	L1c2	African
112	1		189 223 265C 278 286G 294 311 343T 360	L1c2	African
113	1		193 213 223 239 278 294 309 390	L2a1β1	African
114	1		223 292 320	L3e2	African
115	1		86 149T 152A 223 248 320 355	L3e2	African
116	1	2	209 223 311	L3f	African
		76	Several^c^	Several^c^	African
	172	107			

^a^The nucleotide positions considered for the analyses were from 16,050 to 16,391. Note that a value of 16,000 has been subtracted from each nucleotide position to make this column easier to read. Sequences were aligned with the revised reference sequence (rCRS; Andrews *et al.*, 1999). ^b^Haplogroup nomenclature is that recommended in the literature (see text). Those cases in which HVS-I information alone did not allow the identification of specific haplogroups were classified based on probabilities. ^c^Complete information about the African lineages observed in Porto Alegre blacks can be found in Hünemeier *et al.* (2007).

**Table 3:** Estimates of parental contribution based on mtDNA and Y-chromosome markers in whites and blacks from Porto Alegre.

Samples and markers	Parental contribution (%)			
	European	Amerindian	African	Reference
White				
mtDNA	69	21	10	Present study
Y-chromosome	99	0	< 1	Present study

Black				
mtDNA	5	16	79	Present study; Hünemeier *et al.* (2007)
Y-chromosome	56	6	38	Present study; Hünemeier *et al.* (2007)

**Table 4:** Estimates of parental contribution using three set of markers and considering persons classified as white or black in different regions of Brazil.

Region and samples	N	Genetic system	Parental contribution (%)^a^			Reference
			European	African	Amerindian	
Whites						
North						
	48	mtDNA^b^	31	15	54	Alves-Silva *et al.* (2000)
	49	Y-chromosome^c^	98	2	0	Carvalho-Silva *et al.* (2001)
	48-2,054	Bi-parental loci^d^	53	3	44	Salzano and Bortolini (2002)
	
Northeast						
	50	mtDNA^b^	34	44	22	Alves-Silva *et al.* (2000)
	49	Y-chromosome^c^	96	4	0	Carvalho-Silva *et al.* (2003)
	64-27,607	Bi-parental loci^d^	72	23	5	Salzano and Bortolini (2002)
	
Southeast						
	99	mtDNA^b^	31	35	34	Alves-Silva *et al.* (2000)
	50	Y-chromosome^c^	96	4	0	Carvalho-Silva *et al.* (2001)
	89-60,270	Bi-parental loci^d^	56	39	5	Salzano and Bortolini (2002)
	
South						
	328	mtDNA^b^	63	16	21	Alves-Silva *et al.* (2000); Marrero *et al.* (2005); present study
	255	Y-chromosome^c^	99	0	< 1	Carvalho-Silva *et al.* (2001); present study
	107-5,527	Bi-parental loci^d^	73	14	13	Salzano and Bortolini (2002)

Blacks						
North						
	270	mtDNA^b^	3	57	40	Silva-Junior *et al.* (2006); Ribeiro-dos-Santos *et al.* (2006)
	ND	Y-chromosome^c^	ND	ND	ND	
	38-482	Bi-parental loci^d^	25	33	42	Salzano and Bortolini (2002)
	
Northeast						
	39	mtDNA^b^	21	69	10	Bortolini *et al.* (1997); Silva-Junior *et al.* (2006)
	89	Y-chromosome^c^	34	64	2	Abe-Sandes *et al.* (2004)
	30-38,898	Bi-parental loci^d^	38	55	7	Salzano and Bortolini (2002)
	
Southeast						
	233	mtDNA^b^	2	89	9	Silva-Junior *et al.* (2006); Gonçalves *et al.* (2007); Hünemeir *et al.* (2007); present study
	288	Y-chromosome^c^	43	56	1	Abe-Sandes *et al.* (2004); Gonçalves *et al.* (2007); Hünemeier *et al.* (2007); present study
	378-33,534	Bi-parental loci^d^	23	77	0	Salzano and Bortolini (2002)
	
South						
	226	mtDNA^b^	4	84	12	Bortolini *et al.* (1997); Hünemeier *et al.* (2007); present study
	55	Y-chromosome^c^	56	38	6	Hünemeier *et al.* (2007); present study
	53-3,236	Bi-parental loci^d^	36	51	13	Salzano and Bortolini (2002)

^a^ND = No data available; ^b^HVS-I sequences and SNPs in the mtDNA codifying region; ^c^SNPs in the non-recombining region of the Y-chromosome; ^d^Classic blood group plus protein polymorphisms.
